# Commentary: Outer Retinal Dysfunction on Multifocal Electroretinography May Help Differentiating Multiple Sclerosis From Neuromyelitis Optica Spectrum Disorder

**DOI:** 10.3389/fneur.2020.00282

**Published:** 2020-04-28

**Authors:** James V. M. Hanson, Sven Schippling, Christina Gerth-Kahlert

**Affiliations:** ^1^Department of Ophthalmology, University Hospital Zurich and University of Zurich, Zurich, Switzerland; ^2^Neuroimmunology and Multiple Sclerosis Research, University of Zurich, Zurich, Switzerland; ^3^Neuroscience Center Zurich (ZNZ), University of Zurich and Federal Institute of Technology (ETH), Zurich, Switzerland

**Keywords:** multiple sclerosis, multifocal electroretinogram, neuromyelitis optica, electrophysiology, bipolar cell

We read with interest the recent manuscript by Filgueiras et al. (1), which presented data collected using multifocal electroretinography (MF-ERG) in patients with multiple sclerosis (MS), neuromyelitis optica spectrum disorder (NMOSD), and control subjects. Given that MF-ERG peak times were slightly shorter (i.e., supernormal) in MS patients relative to both NMOSD patients and controls, the authors interpreted their findings as evidence of excitatory outer retinal dysfunction in MS (1). Whilst this is a potentially exciting conclusion, a degree of caution appropriate to the novelty of the findings is warranted when attempting to interpret these data. In this commentary, we highlight a number of factors which may influence the conclusions drawn from the work of Filgueiras (1).

Evidence to date suggests that full-field ERG peak times in patients with MS are typically delayed (subnormal), with this finding being particularly robust for responses driven, in whole or in part, by the cone system (2–8). While delays to the ERG a-wave have been recorded (2, 4, 5, 8), it appears that delays to the b-wave are more common (2–8). The ERG b-wave is generated primarily in the bipolar cells (9), as is the MF-ERG (10), although both are dependent on functional photoreceptors. Studies employing the MF-ERG in patients with MS are notably fewer in number than those employing full-field ERG, with one study documenting normal MF-ERG findings in an early MS cohort (2) and our group recording responses which were of normal to delayed peak time and normal amplitude (8). In summary, ERG findings in MS patients to date are almost unanimous in showing evidence of subnormal cone-driven bipolar cell function, as evidenced by delayed peak times; MF-ERG evidence to date is sparse, but nevertheless consistent with normal or subnormal, rather than supernormal, cone-driven bipolar function.

How, then, can we interpret the findings of Filgueiras et al. (1) suggesting supernormal bipolar function in MS patients in the light of this pre-existing literature, and are there any factors which may account for this apparent discrepancy? In attempting to reconcile their findings with previous work from our group (8), Filgueiras suggested that our findings may be less reliable due to the use of manufacturers' normative databases from our electrophysiology device for comparison, rather than control data acquired on-site using the same conditions as for our patient cohort. However, this suggestion is incorrect: as we stated in our manuscript (8), all normative data had been previously acquired on-site using the same devices used for the MS patients. Additionally, electrophysiological protocols were identical for MS patients and for acquisition of normative data. This can therefore be discounted as a possible reason for the discrepancy between the two studies.

In contrast, however, the parameters analyzed differed between the two investigations. These differences included the MF-ERG waveform components and retinal areas analyzed. The exclusion of R5 from analysis by Filgueiras et al. (1), in order to facilitate comparison with perimetric data, may be particularly relevant given that we recorded R5 P1 peak times which were significantly delayed in MS patients relative to normative data (8). The analysis of different retinal areas makes it challenging to compare the results of the two studies directly.

Likely the most important factor distinguishing the recent work of Filgueiras et al. (1) from other studies is the choice of analyses employed. Despite analyzing numerous outcome measures, the authors did not correct their published *p*-values for multiple testing. Such corrections are essential when testing multiple hypotheses because the risk of a type 1 (false positive) error increases with the number of statistical comparisons; in other words, the greater the number of hypotheses tested, the greater the possibility of a “statistically significant” result occurring by chance, unless *p*-values are corrected. There are many methods of making such corrections [e.g., the method of Benjamini and Hochberg (11)], but the simplest (12) is Bonferroni correction (13). This corrects *p*-values by dividing the required level of statistical significance by the number of hypotheses to be tested. For example, if five hypotheses are tested, then only *p*-values < (0.05/5=) 0.01 would reach significance.

Filgueiras analyzed the amplitude and peak time of both N1 and P1 components of the MF-ERG over three retinal areas (R1-R2, R3-R4, R1-R4) and employed a significance level of 0.05 (1). Eyes were categorized as MS or NMOSD, in addition to having a history of optic neuritis, and compared with healthy control eyes. Therefore, a total of 48 hypotheses (4 MF-ERG parameters^*^3 retinal areas^*^4 categories) were tested. Applying Bonferroni correction with a significance level of 0.05, only *p*-values < (0.05/48=) 0.001 would reach statistical significance. As the relevant *p*-values were not provided, but merely categorized as being <0.05 or <0.01 [Table 3, (1)], it remains unclear whether any of the purportedly “significant” findings actually reached the specified level of statistical significance; if not, then the pattern of results would be identical (2) or comparable to (8) previously published work.

Finally, we wish to reiterate that Bonferroni correction is only one of many potential ways of addressing the statistical issues raised by multiple hypothesis testing, and moreover is known to be low-powered and statistically conservative (12) [conversely, findings which remain significant after Bonferroni correction of *p*-values are highly likely to be robust in nature (14)]. We have employed this method here simply to illustrate the need for caution when attempting to contextualize the results documented by Filgueiras et al. (1) without reanalyzing the dataset. Inhibitory bipolar cell dysfunction in patients with MS is now well-documented (as discussed above), however similarities or differences between findings in the central and peripheral retinae are yet to be definitively elucidated, as is the utility and clinical relevance of this dysfunction. With regard to potential excitatory bipolar dysfunction in MS, as Filgueiras and colleagues correctly state in their manuscript (1), further studies are required. For these reasons, the currently available body of evidence appears insufficient to support the existence of supernormal retinal bipolar cell function in patients with MS.

## Author Contributions

JH conceived and wrote the manuscript. SS and CG-K revised and approved the manuscript.

## Conflict of Interest

The authors declare that the research was conducted in the absence of any commercial or financial relationships that could be construed as a potential conflict of interest.

## References

[1] Filgueiras TG Oyamada MK Preti RC Apóstolos-Pereira SL Callegaro D Monteiro MLR. Outer retinal dysfunction on multifocal electroretinography may help differentiating multiple sclerosis from neuromyelitis optica spectrum disorder. Front Neurol. (2019) 10:928. 10.3389/fneur.2019.00928 31507527 PMC6718638

[2] Gundogan FC Demirkaya S Sobaci G. Is optical coherence tomography really a new biomarker candidate in multiple sclerosis?–A structural and functional evaluation. Invest Ophthalmol Vis Sci. (2007) 48:5773–81. 10.1167/iovs.07-0834 18055831

[3] Sriram P Wang C Yiannikas C Garrick R Barnett M Parratt J. Relationship between optical coherence tomography and electrophysiology of the visual pathway in non-optic neuritis eyes of multiple sclerosis patients. PLoS ONE. (2014) 9:e102546. 10.1371/journal.pone.0102546 25166273 PMC4148263

[4] You Y Graham EC Shen T Yiannikas C Parratt J Gupta V. Progressive inner nuclear layer dysfunction in non-optic neuritis eyes in MS. Neurol Neuroimmunol Neuroinflamm. (2018) 5:e427. 10.1212/NXI.0000000000000427 29259999 PMC5732006

[5] You Y Zhu L Zhang T Shen T Fontes A Yiannikas C. Evidence of muller glial dysfunction in patients with aquaporin-4 immunoglobulin G-positive neuromyelitis optica spectrum disorder. Ophthalmology. (2019) 126:801–10. 10.1016/j.ophtha.2019.01.016 30711604

[6] Forooghian F Adamus G Sproule M Westall C O'Connor P. Enolase autoantibodies and retinal function in multiple sclerosis patients. Graefes Arch Clin Exp Ophthalmol. (2007) 245:1077–84. 10.1007/s00417-006-0527-8 17219105

[7] Forooghian F Sproule M Westall C Gordon L Jirawuthiworavong G Shimazaki K. Electroretinographic abnormalities in multiple sclerosis: possible role for retinal autoantibodies. Doc Ophthalmol. (2006) 113:123–32. 10.1007/s10633-006-9022-0 16972082

[8] Hanson JVM Hediger M Manogaran P Landau K Hagenbuch N Schippling S Outer retinal dysfunction in the absence of structural abnormalities in multiple sclerosis. Invest Ophthalmol Vis Sci. (2018) 59:549–60. 10.1167/iovs.17-22821 29372255

[9] McCulloch DL Marmor MF Brigell MG Hamilton R Holder GE Tzekov R. ISCEV Standard for full-field clinical electroretinography (2015 update). Doc Ophthalmol. (2015) 130:1–12. 10.1007/s10633-014-9473-7 25502644

[10] Hood DC Frishman LJ Saszik S Viswanathan S. Retinal origins of the primate multifocal ERG: implications for the human response. Invest Ophthalmol Vis Sci. (2002) 43:1673–85. 11980890

[11] Benjamini Y Hochberg Y Controlling the false discovery rate: a practical and powerful approach to multiple testing. J R Stat Soc Ser B. (1995) 57:289–300. 10.1111/j.2517-6161.1995.tb02031.x

[12] Bender R Lange S. Adjusting for multiple testing–when and how? J Clin Epidemiol. (2001) 54:343–9. 10.1016/S0895-4356(00)00314-0 11297884

[13] Dunn OJ Multiple comparisons among means. J Am Stat Assoc. (1961) 56:52–64. 10.1080/01621459.1961.10482090

[14] VanderWeele TJ Mathur MB. Some desirable properties of the Bonferroni Correction: is the Bonferroni Correction really so bad? Am J Epidemiol. (2019) 188:617–8. 10.1093/aje/kwy250 30452538 PMC6395159

